# A multi-filter enhanced genetic ensemble system for gene selection and sample classification of microarray data

**DOI:** 10.1186/1471-2105-11-S1-S5

**Published:** 2010-01-18

**Authors:** Pengyi Yang, Bing B Zhou, Zili Zhang, Albert Y Zomaya

**Affiliations:** 1 School of Information Technologies (J12), The University of Sydney, NSW 2006, Australia; 2 NICTA, Australian Technology Park, Eveleigh, NSW 2015, Australia; 3 Faculty of Computer and Information Science, Southwest University, CQ 400715, PR China; 4 School of Information Technology, Deakin University, VIC 3217, Australia; 5 Sydney Bioinformatics, The University of Sydney, NSW 2006, Australia; 6 Centre for Mathematical Biology, The University of Sydney, NSW 2006, Australia; 7 Centre for Distributed and High Performance Computing, The University of Sydney, NSW 2006, Australia

## Abstract

**Background:**

Feature selection techniques are critical to the analysis of high dimensional datasets. This is especially true in gene selection from microarray data which are commonly with extremely high feature-to-sample ratio. In addition to the essential objectives such as to reduce data noise, to reduce data redundancy, to improve sample classification accuracy, and to improve model generalization property, feature selection also helps biologists to focus on the selected genes to further validate their biological hypotheses.

**Results:**

In this paper we describe an improved hybrid system for gene selection. It is based on a recently proposed genetic ensemble (GE) system. To enhance the generalization property of the selected genes or gene subsets and to overcome the overfitting problem of the GE system, we devised a mapping strategy to fuse the goodness information of each gene provided by multiple filtering algorithms. This information is then used for initialization and mutation operation of the genetic ensemble system.

**Conclusion:**

We used four benchmark microarray datasets (including both binary-class and multi-class classification problems) for concept proving and model evaluation. The experimental results indicate that the proposed multi-filter enhanced genetic ensemble (MF-GE) system is able to improve sample classification accuracy, generate more compact gene subset, and converge to the selection results more quickly. The MF-GE system is very flexible as various combinations of multiple filters and classifiers can be incorporated based on the data characteristics and the user preferences.

## Background

Feature selection is an important process for high dimensional data analysis. With the advancement of new high-throughput bio-technologies, feature selection quickly found its use in the analysis of the massive quantity of generated data [1]. The gene selection in microarray data is one of such crucial applications because microarray datasets inherently have high feature-to-sample ratio, i.e., several thousands of features (genes) with only a few dozen of samples [2]. To identify biologically significant biomarkers and to improve the ability in new case diagnosis, robust and scalable feature selection methods play a critical role.

**Figure 1:**
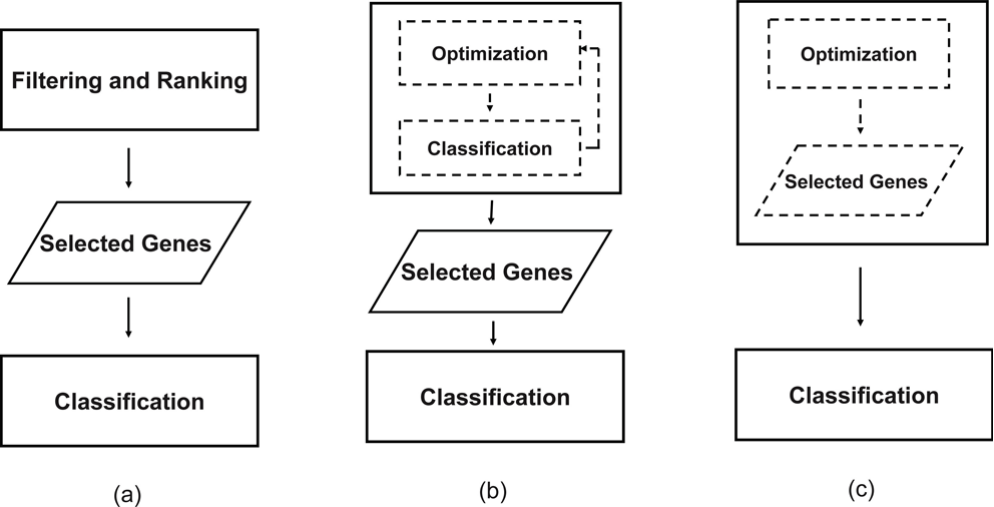
**Different types of feature selection algorithms**. (a)Filter approach (b)Wrapper approach (c)Embedded approach.

Currently, three major types of feature selection models have been intensively utilized for gene selection and dimension reduction in microarray data. The first type is known as "filter" approach. Typically, filtering algorithms do not optimize the classification accuracy of the classifier directly, but attempt to select genes with certain kind of evaluation criterion. Examples are χ^2^-statistic [3], *t*-statistic [4], ReliefF [5], Information Gain, and Gain Ratio [6]. With the filter approach the gene selection process and the classification process are thus separated, as shown in Figure 1(a). The advantages are that the algorithms are often fast and the selected genes are better generalized to unseen data classification. However, to ignore the effects of the selected gene subset on the performance of the classifier may cause crucial information being lost for accurate sample discrimination and target gene identification [7]. More importantly, filtering algorithms often treat each gene independently. Nevertheless, genes are commonly connected by various bio-pathways and functioning as groups. Such one gene at a time methods often miss important bio-pathway information.

Different from filters, the "wrapper" approach evaluates the selected gene subset according to their power to improve sample classification accuracy [7]. The classification thus is "wrapped" in the gene selection process, as depicted in Figure 1(b). Classical wrapper algorithms include forward selection and backward elimination [8]. Recently, evolutionary based algorithms such as Genetic Algorithm (GA) and Evolution Strategy (ES) have been introduced as more advanced wrapper algorithms for the analysis of microarray datasets [9-12]. Unlike classical wrappers which select genes incrementally [13], GA selects genes nonlinearly by creating gene subset randomly. Furthermore, GA is efficient in exploring large searching space for solving combinatorial problems [14]. This makes it a promising solution for gene selection in microarray data. Nevertheless, wrapper approaches like GA have long been criticized for suffering from overfitting [1] because an inductive algorithm is usually used as the sole criterion in gene subset evaluation. In other words, the use of a given inductive algorithm as the sole optimization guide leads the system to seek for high classification accuracy on training data blindly which may give poor generalization property on unseen data classification.

The third group of selection scheme is known as embedded approaches, which use the inductive algorithm itself as the feature selector as well as classifier. As illustrated in Figure 1(c), feature selection is actually a by-product of the classification process. Example are classification trees such as ID3 [15] and C4.5 [16]. However, the drawback of embedded methods is that they are generally greedy based [8], using only top ranked genes to perform sample classification in each step while an alternative split may perform better. Furthermore, additional steps are required to extract the selected genes from the embedded algorithms.

To address the drawbacks of each method while attempt to take advantage of their strengthes, various hybrid algorithms have been proposed. In [17], Yang et al. pointed out that no one filter algorithm is universally optimal and there is seldom any basis or guidance to the choice of a particular filter for a given dataset. They proposed a hybrid method which synthesizes several different filters using a special designed distance. Their experimental results indicate that including multiple source of information is an advantage in improving prediction accuracy. However, this approach, too, did not incorporate classification information which could be very useful in obtaining more accurate sample classification result.

Since relying on a single classifier often gives bias and overfitted classification results, designing multiple classifier system to weigh the classification hypotheses also received much attention [18,19]. To incorporate the benefits of GA in evaluating features by groups and in extracting nonlinear relationship from associated features, we recently proposed a genetic ensemble (GE) framework for feature selection [20]. By applying multiagent techniques for hybrid system composition under the proposed genetic framework, we found a GE combination, which is superior to many alternatives in the context of microarray data analysis [21]. In that system multiple classifiers were applied to evaluate the goodness of gene subsets, and the system works in an iterative way, collecting multiple gene subsets as candidate sample classification profiles. The preliminary experimental results suggest that the GE system is able to improve the sample classification accuracy and the reproducibility of the gene selection results which is often overlooked [22].

To further improve the generalization property of the selected genes and gene subsets on unseen data classification, in this study, we incorporate multiple filtering algorithms into the GE system. This more advanced system is named the multi-filter enhanced genetic ensemble system, or MF-GE for short. A novel mapping strategy for multiple filtering information fusion is developed to fuse the evaluation scores from multiple filters, and this strategy is incorporated into the GE system for gene selection and classification. Thus the initialization and mutation processes of the original genetic ensemble system is governed by the knowledge generated from multiple filtering algorithms.

We compare the MF-GE system with the original GE system and the GA/KNN hybrid proposed by Li et al. [9] which is similar to GE except that the optimization is guided by *k*-nearest neighbor classifiers. Also, Gain Ratio filtering algorithm (which is commonly employed for gene selection of microarray datasets) is used as an additional yardstick. We found that this improved system is able to produce higher classification accuracy, generate more compact gene subset, and converge to the selection results more quickly. More importantly, the proposed multi-filter mapping component and the genetic ensemble component are very flexible, allowing any filters/classifiers with new capabilities to be added to the system and those no longer used to be deleted from the system based on the data requirements or user preferences.

## Methods

### The MF-GE hybrid approach

#### System overview

**Figure 2:**
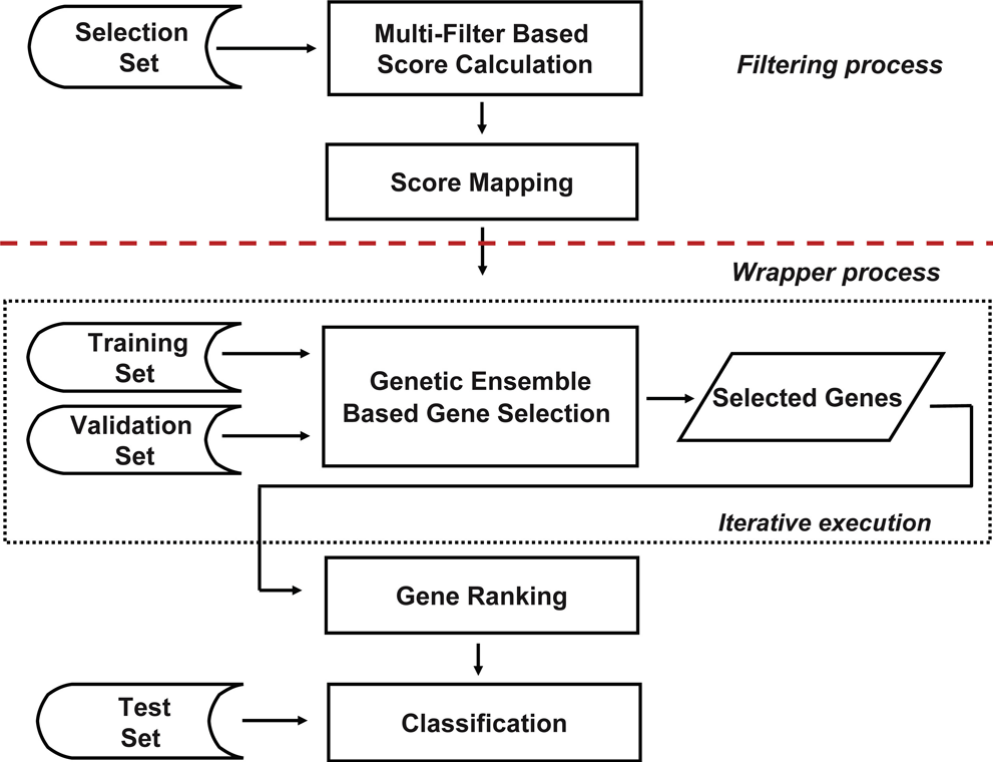
**The flow chart of the MF-GE hybrid system for gene selection and classification of microarrays**.

A flow chart of the proposed MF-GE hybrid system is illustrated in Figure 2. In this system the gene selection process is sequentially divided into two phases, i.e., "filtering process" and "wrapper process". In the filtering process, multiple filtering algorithms are applied to give scores for each candidate gene in the microarray dataset. The scores of each gene are then integrated for wrapper process. In the wrapper process, the genetic ensemble algorithm is used to select discriminative genes using the information provided by the filtering process. The detail of this genetic ensemble algorithm is described in [20,21]. Basically, a multiple objective GA (MOGA) is utilized as the gene combination search engine while an ensemble of the classifiers is used as the gene subsets evaluation component to provide feedback for gene subsets optimization. The algorithm executes iteratively, collecting multiple gene subsets. The final collections are ranked and the top genes are used for sample classification.

An intermediate step called "score mapping" serves as the synergy between the filtering process and the wrapper process. It is described in details in the next subsection.

#### Multi-filter score mapping

**Figure 3:**
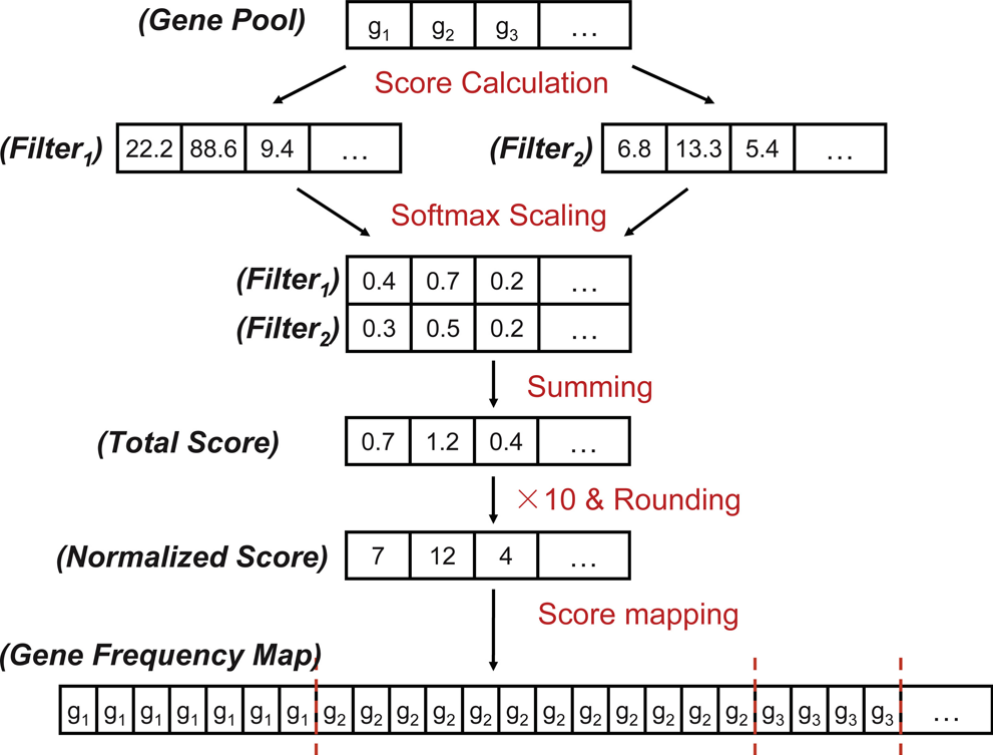
**An example of multiple filter score mapping strategy for evaluation information fusion**.

Traditionally, filtering algorithms select differential genes independently for the classification process. However, such information could be beneficial if appropriately integrated into the wrapper procedure. To fuse the evaluation information from multiple filtering algorithms, we developed a multi-filter score mapping strategy which serves as the connection between the filtering process and the wrapper process. An example of this mapping process with two filters is depicted in Figure 3.

The process starts by calculating scores for each candidate gene with different filtering algorithms. The evaluation scores obtained from different filtering algorithms are then integrated. One issue in integrating multiple scores is that different filtering algorithms often provide evaluation scores with different scales. In order to combine the evaluation results of multiple filters, we must transform the evaluation scores into a common scale. Therefore, the softmax scaling process is adopted to squash the gene evaluation results of each filtering algorithm into the range of 0[1]. The calculation is as follows:

in which

where  is the average expression value of the *k*th gene among all samples, *σ*_*k *_is the standard deviation of the *k*th gene among all samples, and  is the transformed value of *x*_*ik *_which denotes the expression value of the *k*th gene in sample *i*.

After the softmax scaling process, the evaluation scores with different filtering algorithms are summed up to a set of total score which indicates the overall score of each gene under the evaluation of multiple filtering algorithms. The total scores are then timed with 10 and rounded into integer. Those with scores smaller than 1 are set to score of 1 to make sure all candidate genes are included in the wrapper selection process. The final step is the score-to-frequency mapping step which transfers the given integer of each gene into the appearance frequency of this gene in the transferred candidate gene pool (we call it a gene frequency map). The random processes of "chromosome" initialization and the "chromosome mutation" of the genetic ensemble system are then conducted based on this gene frequency map.

It is readily noticed that genes with higher overall evaluation scores will appear in the gene frequency map more frequently, thus, will have a better chance to be chosen in the initialization step and the mutation step. In this way, multiple filter information is fused into the gene selection process, which helps to integrate information of data characteristics from different aspects.

### Filters and classifiers

#### Filter components

In this subsection, we introduce five filtering algorithms incorporated in our MF-GE hybrid system for experiments. All these filtering algorithms have been routinely applied for gene selection of microarray data.

### χ^2^-statistic

When used for gene evaluation, χ^2^-statistic can be considered as to calculate the occurrence of a particular value of a gene and the occurrence of a class associated with this value. Formally, the discriminative power of a gene is quantified as follows:

where *c*_*i*_, (*i *= 1, ..., *m*) denotes the possible classes of the samples from a dataset, while *g *is the gene that has a set of possible values denoted as *V *. *N*(*g *= *v*, *c*_*i*_) and *E*(*g *= *v*, *c*_*i*_) are the observed and the expected co-occurrence of *g *= *v *with the class *c*_*i*_, respectively.

### ReliefF

ReliefF is a widely used filtering algorithm. In microarray data classification context, the algorithm selects genes that have high resolution distinguishing samples which have similar expression patterns. The formula used by ReliefF to compute the weight or "importance" of a gene *g *is as follows:

where *diff*(*g*, *S*_1_, *S*_2_) calculates the difference between the values of the gene *g *for two samples (*S*_1 _and *S*_2_),  denotes the *i*th randomly selected samples from the dataset, while *S*_*d *_and *S*_*s *_denote nearest sample from a different class to  and nearest sample from the same class to , respectively. *N*(.) is a normalization function which keeps the value of *W*(*g*) to be in the interval [-1, 1].

### Symmetrical Uncertainty

The Symmetrical Uncertainty method evaluates the worth of an gene by measuring the symmetrical uncertainty with respect to the sample class [23]. Each gene is evaluated as follows:

where *H*(.) is the information entropy function. *H*(*class*) and *H*(*g*) give the entropy values of the class and a given gene, while *H*(*class*|*g*) gives the entropy value of a gene with respect to the class.

### Information Gain

Information Gain is a statistic measure often used in nodes selection for decision tree construction. It measures the number of bits of information provided in class prediction by knowing the value of feature [3]. Let *c*_*i *_belong to a set of discrete classes (1, ..., m). *V *be the set of possible values for a given gene *g*. The information gain of a gene *g *is defined as follows:

### Gain Ratio

The final filtering algorithm used in the hybrid system is Gain Ratio. Gain Ratio incorporates "split information" of features into Information Gain statistic. The "split information" of a gene is obtained by measuring how broadly and uniformly it splits the data [24]. Let's consider again a microarray dataset has a set of classes denoted as *c*_*i*_, (*i *= 1, ..., *m*), and each gene *g *has a set of possible values denoted as *V *. The discriminative power of a gene *g *is given as:

in which:

where *S*_*v *_is the subset of *S *of which gene *g *has value *v*.

It is clear that each algorithm uses a different criterion in evaluating the worth of the candidate genes in microarray datasets. When combined, candidate genes are assessed from many different aspects.

#### Classifier components

Ensemble of classifiers has recently been suggested as a promising measure to overcome the limitation of individual classifier [25]. In our previous study, we demonstrated that if combined properly, multiple classifiers can achieve higher sample classification accuracy and more reproducible feature selection results [20]. Therefore, selecting classification algorithms and developing suitable integration strategies are the key to a successful ensemble. What characteristics should we promote in the ensemble construction? The basic concerns are that they should be as accurate and diverse as possible [26], and the individual classifiers should be relatively computationally efficient. With these criteria in mind, we evaluated different composition under the genetic architecture within a multiagent framework [21]. A hybrid of five classifiers, namely, decision tree (DT), random forest (RF), 3-nearest neighbors (3NN), 7-nearest neighbors (7NN), and naive bayes (NB) is identified to be better in terms of sample classification and stability than many alternatives. Furthermore, two integration strategies, namely, *blocking *and *majority voting *have been employed for ensemble construction.

The blocking strategy optimizes the target gene subset by improving the sample classification accuracy using the whole ensemble rather than one specific inductive algorithm. This formulation adds multiple test conditions into the algorithm, and the gene subset optimized under this criterion will not tie to any specific classifier, but have a more generalization nature. Moreover, genes selected with this strategy are more likely to have real relevance to the biological trait of interest [27]. The majority voting combines multiple classifiers and tries to optimize the target feature set into a superior set in producing high consensus classification [28]. This part of the function promotes the selected genes in creating diverse classifiers implicitly, which in turn leads to the high sample classification accuracy [29].

The fitness functions derived from blocking (*fitness*_*b*_(*s*)) and majority voting (*fitness*_*v*_(*s*)) are defined as follows:

and

where *k *is the size of the majority voting *V*_*k*_(.), *h*_*i*_(*s*), (*i *= 1, ..., *L*) is the classification hypothesis generated by classifier *i *in the ensemble while classifying dataset using gene subset *s*, *y *is the class label of samples, and *BC*(.) is the balanced classification accuracy which is calculated as follows:

and

where *Se*_*j *_is the sensitivity value calculated as the percentage of the number of true positive classification () of samples in class *j*, *N*_*j *_denotes the total number of samples in class *j*, and *m *is the total number of classes.

Finally, the fitness function of the MOGA is defined as follows:

where the empirical coefficients *w*_1 _and *w*_2 _specify the contribution weights of each term.

## Results and discussion

This section describes the experimental settings and presents the experimental results.

### Experimental settings

#### Datasets and data pre-processing

We gathered four benchmark microarray datasets for system evaluation, including binary-class and multi-class classification problems. Table 1 summarizes each dataset.

**Table 1:** Microarray datasets for evaluation

Name	Leukemia	Colon	Liver	MLL
**Ref**.	[30]	[31]	[32]	[33]
# Sample	72	62	157	72
# Gene	7129	2000	20983	12582
# Class	2	2	2	3
C1	ALL: 47	TUM: 40	HCC: 82	ALL: 24
C2	AML: 25	NOR: 22	NON: 75	MLL: 20
C3				AML: 28

The "Leukemia" dataset [30] investigates the expression of two different subtypes of leukemia (47 ALL and 25 AML), and the "Colon" dataset [31] contains expression patterns of 22 normals and 40 cancerous tissues. The "Liver" dataset [32] has 82 samples labeled as Hepatocellular carcinoma (HCC) and other 75 samples labeled as Non-tumor. The task for these three datasets is to identify a small group of genes which can distinguish samples from two classes. The "MLL" dataset [33] provides a multi-classes classification problem. The task is to discriminate each class using a selected gene profile. These four datasets cover the general situations in gene selection and sample classification of microarray datasets.

In order to objectively differentiate and compare the power of different feature selection algorithms, we applied a double cross validation process. That is, each dataset is partitioned by an external cross validation and an internal cross validation. The gene selection process is conducted on the internal cross validation sets while the external cross validation sets are used for evaluating the selection results.

Data normalization and pre-processing are of great importance and can have heavy influence on the success of the overall analysis. Based on the previous studies, only a few dozens of genes (or even only a few genes) are needed for sample classification in general [34,35]. Therefore, for each microarray dataset 200 genes are pre-filtered from the external train sets, which are then suitable for follow up precise gene selection. Specifically, we apply the following pre-processing steps:

1. Standardize the gene expression levels of the dataset with the mean of 0 and the variance of 1.

2. Normalize the gene expression levels of the dataset into [0, 1].

3. Split each dataset into external train sets and external test sets with an external 3-fold stratified cross validation.

4. Rank each gene in the external train sets with the between-group to within-group sum of square ratio (BSS/WSS) [36].

5. Pre-filter the external train sets by selecting the top 200 genes from the ranking list.

6. Split the external train sets into internal train sets and internal test sets with an internal 3-fold stratified cross validation.

The gene score calculation is conducted by using the internal train sets while the wrapper selection is performed using internal train sets and internal test sets collaboratively. The external test sets are reserved for the evaluation of the selected genes on unseen data classification, and are excluded from pre-filtering as well as the gene selection processes.

#### Implementation

For the genetic ensemble component, a set of initial tests is conducted to evaluate different parameter configurations, from which parameter values are chosen and fixed for the latter experiments.

The iteration of the genetic ensemble procedure is set to 100. Within each iteration, the population size of GA is 100. These 100 populations are divided into two niches each with 50, and is evolved separately. After every 10 generations, the favorite chromosomes from each niche are exchanged to the other. The probability of crossover *p*_*c *_is 0.7. A novel mutation strategy is implemented to allow multiple mutations, that is, when a single mutation happened (with the probability of 0.1) on a chromosome, another single point mutation may happen on the same chromosome with the probability of 0.25 and so on. The selection method is the tournament selection with the candidate size of 3, and the contribution weights of *w*_1 _and *w*_2 _are set to 0.5. Lastly, the termination condition for each iteration is either that the termination generation of 100th is reached or the similarity of the population converges to 90%. Table 2 summarizes the parameter settings.

**Table 2:** Genetic ensemble settings

Parameter	Value
Fitness Function	Multi-Objective
Iteration	100
Population Size	100
Niche	2
Chromosome Size	15
Termination	Multiple Conditions
Selection	Tournament Selection (3)
Crossover	Single Point (0.7)
Mutation	Multi-Point (0.1 & 0.25)
Contribution Weight	*w*_1 _= 0.5, *w*_2 _= 0.5

In our parameter tuning experiments, the average gene subset size is within 2 to 10. Thus, the GA chromosome is represented as a string of size 15. In chromosome coding, each position is used to specify the *id *of a selected gene or assigned a "0" to denote no gene is selected at the current position. This gives a population of gene subsets of different sizes with a maximum of 15.

Classifiers and filters are created by using Waka - a machine learning suite which provides the implementation of various popular machine learning and data mining algorithms [23]. In specific, J48 algorithm is used to create classification tree. Random forest algorithm with size of 7 trees is applied, while *k*-nearest neighbor and naive bayes classifiers are adopted with default parameters. Each filtering algorithm is provoked for evaluation of each candidate gene and integrated from our main code through the class API of Waka.

The GA/KNN code were downloaded from the author's web site [37]. The chromosome length of 15, the iteration of 1000, and the majority voting with *k *= 3 of the *k*NN were used. For each dataset, GA/KNN requires a pre-specified selection threshold of cut-off. Therefore, different thresholds were used according to their classification power on different datasets.

## Results

The first set of experiments is set out to compare the classification accuracy of the selected gene sets from MF-GE hybrid with GE, GA/KNN, and Gain Ratio filter algorithm. Instead of trying to achieve the highest classification accuracy, we focus on differentiating the classification power of different gene selection algorithms. The ranking and classification of each dataset are repeated 5 times and each time the top 5, 10, 15, and 20 genes are used for sample classification. We report the average of the classification results.

The evaluation results obtained from different microarray datasets are depicted in Tables 3, 4, 5, 6, respectively. In each table, the classification results using each individual classifier as well as the mean and the majority voting of them are listed. It is easy to see that the MF-GE system has a higher average classification accuracy for all datasets. For example, 1.20%, 1.33%, 0.75%, and 1.85% improvements of mean over the original GE (which is the second best in average over all datasets) are obtained using the MF-GE system for Leukemia, Colon, Breast, and MLL, respectively. Given the fact that the GE part of these two algorithms are the same, the natural explanation of the improvement is attributed to the fusion of multiple filter information.

**Table 3:** Classification comparison of different gene ranking algorithms using Leukemia dataset

Dataset	Classifier	Algorithm			
		
		Gain Ratio	GA/KNN	GE	MF-GE
Leukemia	C4.5	87.41	78.55 ± 2.96	83.04 ± 1.56	84.51 ± 2.53
	Random Forests	92.59	91.75 ± 0.99	90.82 ± 1.87	92.35 ± 0.70
	3-Nearest Neighbor	91.16	93.74 ± 1.27	94.30 ± 1.73	95.48 ± 0.95
	7-Nearest Neighbor	83.10	89.43 ± 1.10	90.45 ± 2.04	90.86 ± 1.26
	Naive Bayes	92.78	90.28 ± 1.33	96.20 ± 0.93	96.27 ± 1.65
	
	Mean	89.41	88.75	90.69	91.89
	Majority Voting	92.45	93.29 ± 1.29	95.33 ± 0.96	96.23 ± 1.26

**Table 4:** Classification comparison of different gene ranking algorithms using Colon dataset

Dataset	Classifier	Algorithm			
		
		Gain Ratio	GA/KNN	GE	MF-GE
Colon	C4.5	71.49	62.43 ± 2.78	73.08 ± 2.77	76.64 ± 1.53
	Random Forests	63.66	73.48 ± 2.09	71.86 ± 2.02	74.35 ± 2.01
	3-Nearest Neighbor	68.02	73.83 ± 1.57	75.43 ± 0.92	77.01 ± 2.09
	7-Nearest Neighbor	65.43	67.62 ± 1.45	68.39 ± 1.76	68.78 ± 2.32
	Naive Bayes	70.61	72.12 ± 1.68	76.46 ± 2.14	75.07 ± 2.38
	
	Mean	68.84	69.90	73.04	74.37
	Majority Voting	70.56	73.37 ± 1.84	75.81 ± 2.00	76.98 ± 1.06

**Table 5:** Classification comparison of different gene ranking algorithms using Liver dataset

Dataset	Classifier	Algorithm			
		
		Gain Ratio	GA/KNN	GE	MF-GE
Liver	C4.5	84.88	88.33 ± 0.94	87.09 ± 0.79	88.19 ± 0.56
	Random Forests	89.65	90.31 ± 1.11	91.87 ± 0.94	93.13 ± 1.18
	3-Nearest Neighbor	87.76	90.46 ± 0.65	93.57 ± 0.57	93.39 ± 0.79
	7-Nearest Neighbor	87.65	89.53 ± 0.56	91.91 ± 0.69	92.54 ± 0.57
	Naive Bayes	89.05	90.85 ± 0.51	92.70 ± 0.67	93.63 ± 0.64
	
	Mean	87.80	89.90	91.43	92.18
	Majority Voting	89.02	91.60 ± 0.36	93.37 ± 0.46	93.80 ± 0.47

**Table 6:** Classification comparison of different gene ranking algorithms using MLL dataset

Dataset	Classifier	Algorithm			
		
		Gain Ratio	GA/KNN	GE	MF-GE
MLL	C4.5	81.87	72.89 ± 2.08	78.27 ± 3.10	81.54 ± 1.67
	Random Forests	83.02	88.07 ± 1.05	88.20 ± 1.41	89.74 ± 0.60
	3-Nearest Neighbor	79.63	88.22 ± 1.30	86.18 ± 1.39	88.14 ± 1.09
	7-Nearest Neighbor	79.63	86.72 ± 1.03	85.02 ± 1.49	86.69 ± 1.98
	Naive Bayes	83.95	89.62 ± 0.67	90.68 ± 1.28	91.50 ± 0.67
	
	Mean	81.62	85.10	85.67	87.52
	Majority Voting	83.88	88.38 ± 0.97	89.02 ± 1.71	91.08 ± 0.96

An apparent question is that whether such improvements with multiple filters justify the additional computational expenses? This question can be answered from two aspects. Firstly, the multi-filter score calculation in the MF-GE system is done only once at the start of the algorithm. This step will not be involved in the genetic iteration and optimization processes. Therefore, it is computationally efficient to incorporate these initial information. Secondly, by closely observing the classification results produced by individual classifiers, we can see that the MF-GE system achieved better classification results in almost all cases than those alternative methods, regardless which inductive algorithm is used for evaluation. Moreover, such improvement is consistent throughout all datasets used for evaluation. This demonstrates that the gene subsets selected by the MF-GE system have a better generalization property and thus are more informative for unseen data classification. From the biological perspective, the selected genes and gene subsets are more likely to have genuine association with the disease of interest. Hence, they are more valuable for future biological analysis.

**Figure 4:**
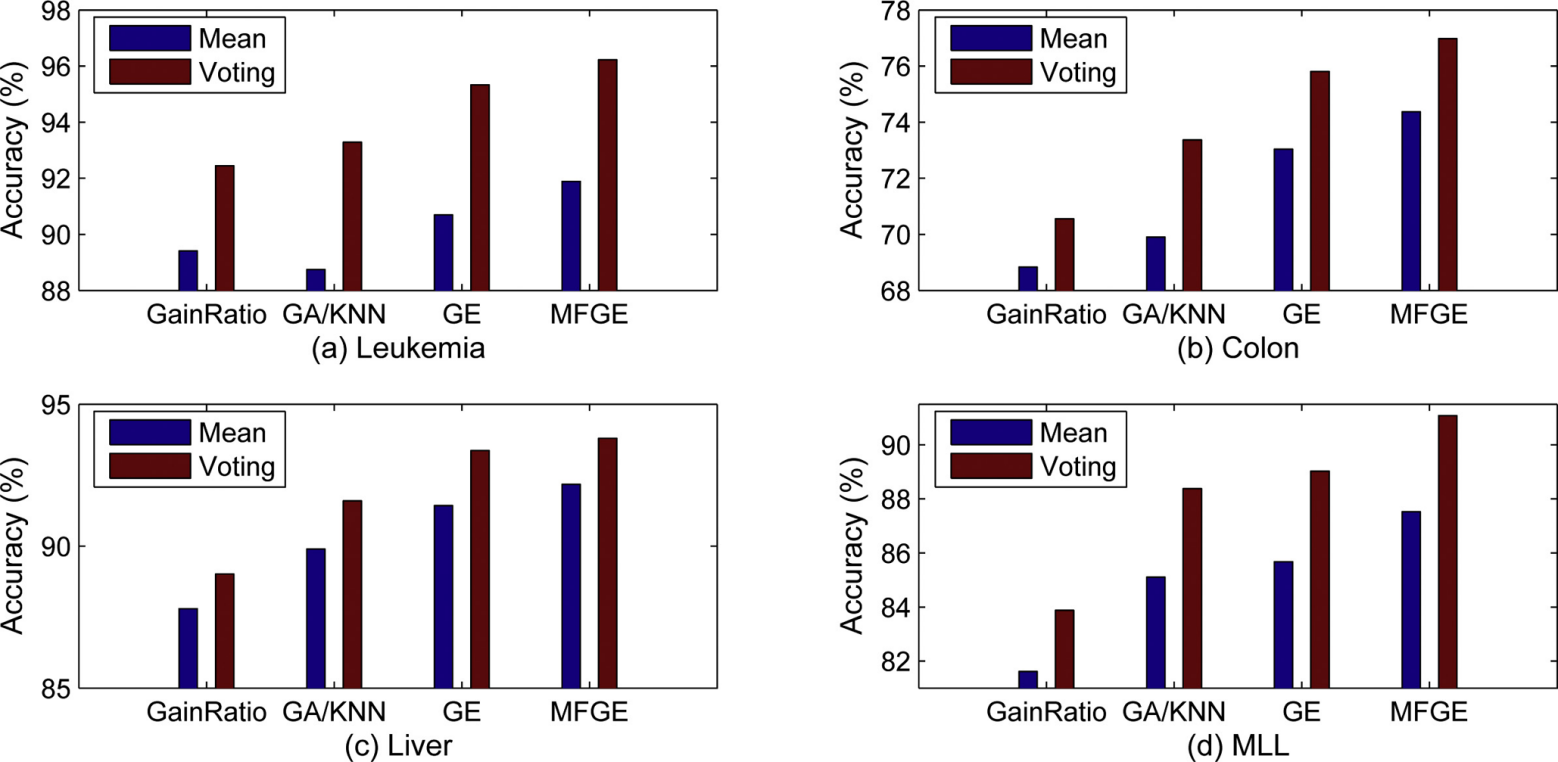
**Sample classification**. The comparison of average classification and majority voting classification of the five classifiers with different gene selection methods in each microarray dataset.

**Figure 5:**
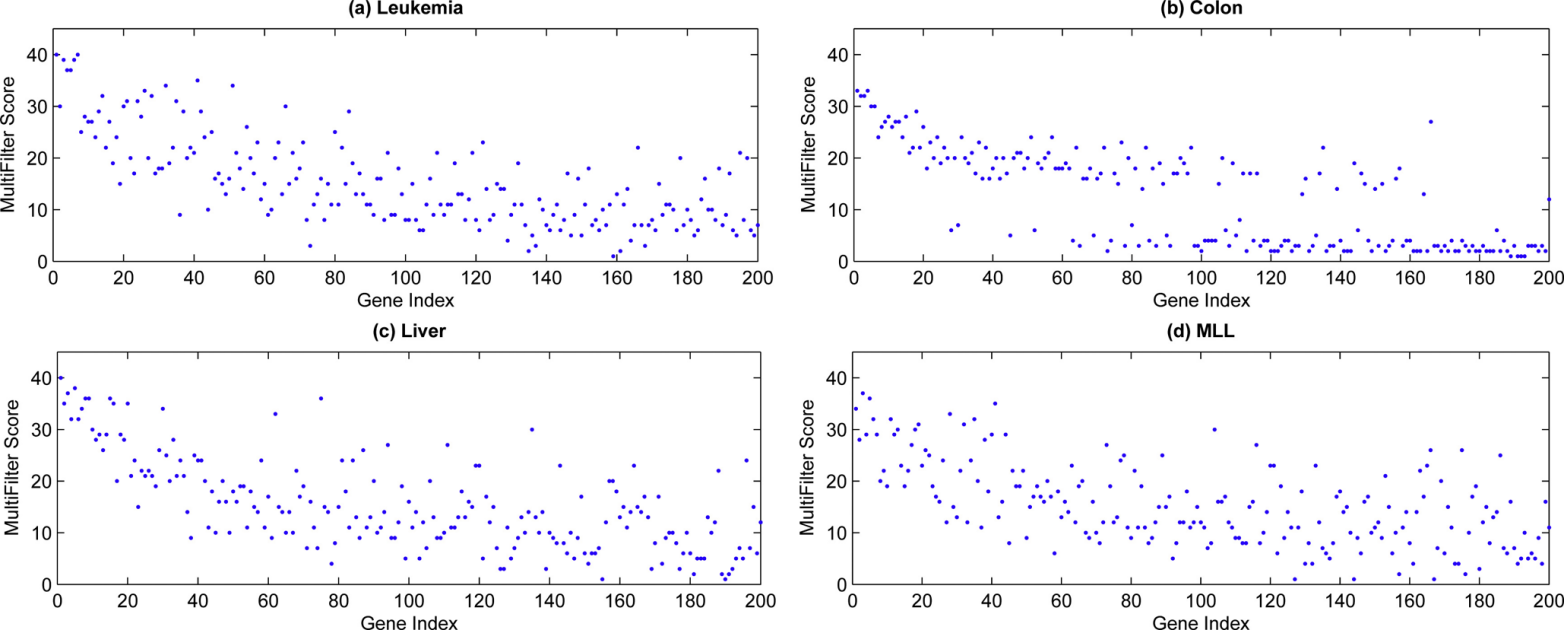
**Multi-filter scores of the 200 genes pre-filtered by BSS/WSS**.

Figure 4 gives the comparison of the mean classification accuracy and the majority voting accuracy of these five classifiers with different gene ranking methods in each microarray dataset. In all cases, integrating classifiers with majority voting gives better classification results than the average of individuals. Therefore, majority voting can be considered as a useful classifier integration method for improving the overall classification accuracy. Figure 5 depicts the multi-filter scores of the 200 genes pre-filtered by BSS/WSS. It is evident that many genes with relatively low BSS/WSS ranking have shown very high multi-filter scores. Interestingly, in colon dataset, genes are fractured into two groups with respect to the multi-filter scores. It is interesting to conduct further study on finding the causality of such inconsistency.

The second set of experiments is conducted to compare the average generation of convergence (termination generation) and the average gene subset size collected in each iteration of the MF-GE and the original GE hybrid. We formulate these two criteria for comparison because the biological relationship with the target disease is more easily identified when the number of the selected genes is small [38], and a shorter average termination generation implies that the method is more efficient in terms of computational time.

**Figure 6:**
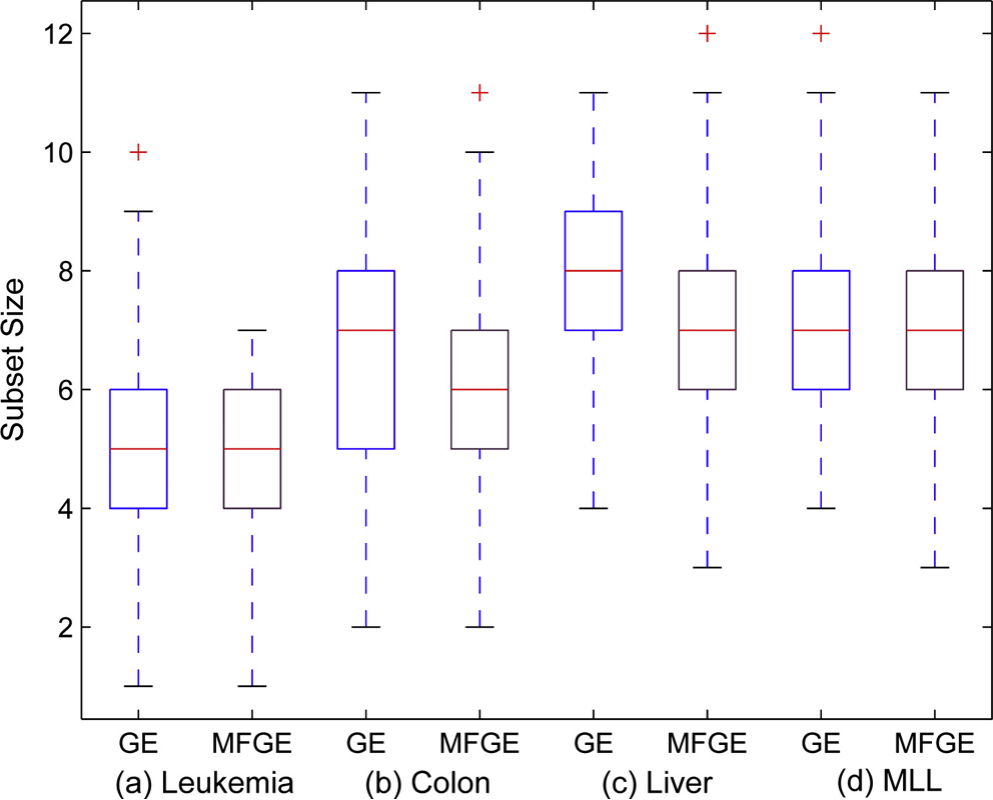
**Average gene subset size selected by GE and MF-GE with each microarray dataset**.

**Figure 7:**
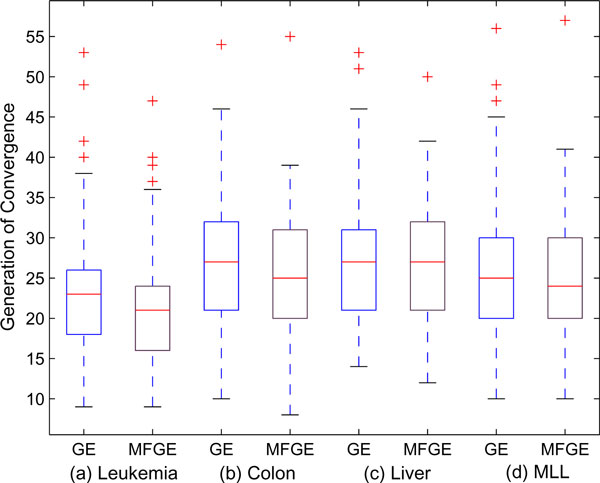
**Average generation of convergence of GE and MF-GE with each microarray dataset**.

As illustrated in Table 7, it is clear that the MF-GE system is capable of converging more quickly while also generating smaller gene subsets. Specifically, the average gene subset size given by MF-GE is about 0.4 to 0.7 of a gene less than those of GE, while the average generation of convergence is about 1 to 2 generations faster. Essentially, the improvement on producing more compact gene subsets is more significant as demonstrated by the *P*-Value of the one tail student *t*-test. The results are also visualized in Figure 6 and Figure 7 using box plotting. One interesting finding is that those figures indicate a dataset-depended relationship, that is, the optimal subset size and the convergence of the genetic component is partially determined by the given dataset. Nevertheless, significant improvements can be achieved by fusion prior data information into the system.

**Table 7:** Generation of convergence & subset size for each dataset using MFGE and GE

Dataset	Comparison Criterion	MF-GE	GE	*P*-Value*
Leukemia	Average Generation of Convergence	21.2	23.4	1 × 10^-2^
	Average Subset Size	4.7	5.4	4 × 10^-3^

Colon	Average Generation of Convergence	25.5	27.1	5 × 10^-2^
	Average Subset Size	6.0	6.6	3 × 10^-3^

Liver	Average Generation of Convergence	27.1	27.4	1 × 10^-1^
	Average Subset Size	7.2	7.7	1 × 10^-3^

MLL	Average Generation of Convergence	25.0	26.1	8 × 10^-2^
	Average Subset Size	6.8	7.2	3 × 10^-2^

*P-Values are calculated using student *t*-test with one tail.

Lastly, in Table 8, we list the top 5 genes with the highest selection frequency of each microarray dataset respectively.

**Table 8:** Top 5 genes with the highest selection frequency of each microarray data

Dataset	Accession Num	Gene Description
Leukemia	X95735_at	Zyxin
	M31523_at	TCF3 Transcription factor 3 (E2A immunoglobulin enhancer binding factors E12/E47)
	Y07604_at	Nucleoside-diphosphate kinase
	M92287_at	CCND3 Cyclin D3
	M27891_at	CST3 Cystatin C (amyloid angiopathy and cerebral hemorrhage)

Colon	Hsa.549	P03001 TRANSCRIPTION FACTOR IIIA
	Hsa.3016	S-100P PROTEIN (HUMAN)
	Hsa.8147	Human desmin gene, complete cds
	Hsa.36689	H. sapiens mRNA for GCAP-II/uroguanylin precursor
	Hsa.6814	COLLAGEN ALPHA 2(XI) CHAIN (Homo sapiens)

Liver	AA232837	Plasmalemma vesicle associated protein (PLVAP)
	AA464192	PDZ domain containing 11 (PDZD11)
	AA486817	Shisa homolog 5 (Xenopus laevis) (SHISA5)
	R43576	Basic leucine zipper nuclear factor 1 (BLZF1)
	H62781	Ficolin (collagen/fibrinogen domain containing lectin) 2 (hucolin) (FCN2)

MLL	33412_at	vicpro2.D07.r Homo sapiens cDNA, 5' end
	1389_at	Human common acute lymphoblastic leukemia antigen (CALLA) mRNA, complete cds
	32847_at	Homo sapiens myosin light chain kinase (MLCK) mRNA, complete cds
	39318_at	H. sapiens mRNA for Tcell leukemia
	40763_at	Human leukemogenic homolog protein (MEIS1) mRNA, complete cds

## Conclusion

Traditionally, filter and wrapper algorithms are treated as competitors in gene selection for data classification. In this study, we embrace an alternative view and attempt to combine them as the building blocks of a more advanced hybrid system. The proposed MF-GE system applied several novel integration ideas to strengthen the advantages of each component while avoiding their weaknesses. The experimental results indicate the followings:

• By fusing evaluation feedbacks of multiple filtering algorithms the system does not only seek for high classification accuracy of training dataset greedily, but takes into consideration other characteristics of the data as well. The overfitting problem can then be circumvented and a better generalization of the selected gene and gene subsets can be achieved.

• By weighing the goodness of each candidate gene from multiple aspects, we reduce the chance of identifying false-positive gene while producing more compact gene subset. This is useful since future biological experiment can be more easily conducted to validate the importance of the selected genes.

• With the use of multiple filtering information, the MF-GE is able to converge more quickly without sacrificing the sample classification accuracy and thus saves computational expenses.

The MF-GE system provides an effective measure for incorporating different algorithm components. It allows any filters or classifiers with new or special capabilities to be added to the system and those no longer useful or inappropriate to be deleted from the system based on the data requirements or user preferences. Finally, the MFGE hybrid system is implemented in Java and is freely available from the project homepage [39].

## Competing interests

The authors declare that they have no competing interests.

## Authors' contributions

PY conceived the study, designed and implemented the algorithms, performed the experiments, and drafted the manuscript. BBZ, ZZ and AYZ drafted part of the manuscript and introduced the problem initially.
